# Irreversible Electroporation of the Pancreas Using Parallel Plate Electrodes in a Porcine Model: A Feasibility Study

**DOI:** 10.1371/journal.pone.0169396

**Published:** 2017-01-04

**Authors:** Steffi J. E. Rombouts, Maarten W. Nijkamp, Willemijn P. M. van Dijck, Lodewijk A. A. Brosens, Maurits Konings, R. van Hillegersberg, Inne H. M. Borel Rinkes, Jeroen Hagendoorn, Fred H. Wittkampf, I. Quintus Molenaar

**Affiliations:** 1 Department of Surgery, University Medical Center Utrecht Cancer Center, Utrecht, the Netherlands; 2 Department of Pathology, University Medical Center Utrecht, Utrecht, the Netherlands; 3 Department of Innovation research, University Medical Center Utrecht, Utrecht, the Netherlands; 4 Department of Cardiology, University Medical Center Utrecht, Utrecht, the Netherlands; University of California at Berkeley, UNITED STATES

## Abstract

**Background:**

Irreversible electroporation (IRE) with needle electrodes is being explored as treatment option in locally advanced pancreatic cancer. Several studies have shown promising results with IRE needles, positioned around the tumor to achieve tumor ablation. Disadvantages are the technical difficulties for needle placement, the time needed to achieve tumor ablation, the risk of needle track seeding and most important the possible occurrence of postoperative pancreatic fistula via the needle tracks. The aim of this experimental study was to evaluate the feasibility of a new IRE-technique using two parallel plate electrodes, in a porcine model.

**Methods:**

Twelve healthy pigs underwent laparotomy. The pancreas was mobilized to enable positioning of the paddles. A standard monophasic external cardiac defibrillator was used to perform an ablation in 3 separate parts of the pancreas; either a single application of 50 or 100J or a serial application of 4x50J. After 6 hours, pancreatectomy was performed for histology and pigs were terminated.

**Results:**

Histology showed necrosis of pancreatic parenchyma with neutrophil influx in 5/12, 11/12 and 12/12 of the ablated areas at 50, 100, and 4x50J respectively. The electric current density threshold to achieve necrosis was 4.3, 5.1 and 3.4 A/cm^2^ respectively. The ablation threshold was significantly lower for the serial compared to the single applications (p = 0.003). The content of the ablated areas differed between the applications: areas treated with a single application of 50 J often contained vital areas without obvious necrosis, whereas half of the sections treated with 100 J showed small islands of normal looking cells surrounded by necrosis, while all sections receiving 4x 50 J showed a homogeneous necrotic lesion.

**Conclusion:**

Pancreatic tissue can be successfully ablated using two parallel paddles around the tissue. A serial application of 4x50J was most effective in creating a homogeneous necrotic lesion.

## Introduction

Stage III pancreatic ductal adenocarcinoma is defined as non-metastasized locally advanced unresectable pancreatic cancer (LAPC) due to arterial encasement or non reconstructable venous involvement. Palliative chemotherapy consisting of gemcitabine monotherapy has been the accepted standard treatment for over a decade. Its survival benefit, however, is marginal with median survival rates of 9.2 to 11.7 months.[1–5] Despite the improvement of survival rates after the advent of FOLFIRINOX, a combination chemotherapy, LAPC still has a poor prognosis. [6]Therefore, local ablative techniques are being explored as a new treatment options for LAPC, in the hope of substantial survival benefit.[7]

The main local ablative techniques currently explored in (randomized) clinical trials are radiofrequency ablation (RFA) and irreversible electroporation (IRE).[7] IRE may have some advantages over RFA since it is a non-thermal procedure that uses high electrical current density to produce nanoscale defects in the cell membrane leading to cell death.[8,9] The lack of a thermal effect with IRE leaves the connective tissue matrix unaffected, which may preserve vascular and ductal structures within the treatment field. [9–13]Both RFA and IRE used today, require placement of needles into and around the tumor.

Using needles entails some limitations. Inserting needles in and around a pancreas tumor obstructing the pancreatic duct leads to the inevitable risk of pancreatic leakage, also known as a pancreatic fistula, occurring in up to 18.8% of patients. [14–17]Second, needle placement may be mandered by excessive tumor mass, an aberrant shape of the tumor or after extensive exploration.[18] Third, as needle track ablation is not possible, a risk of needle track seeding may exist.

To overcome such issues, we investigated the use of a new IRE technique in which two metal plate electrodes, spoon-shaped (‘paddles’) are placed in a parallel fashion outside the pancreatic tissue. This creates an almost homogeneous electrical field without the need for needle insertion into the pancreatic tissue. The aim of this feasibility study was to establish the optimal settings to achieve a successful electroporation ablation in porcine pancreatic tissue, using two paddles.

## Materials and Methods

### Animals and anesthesia

The study was approved by the Animal Ethics Committee of the University Medical Center, Utrecht, the Netherlands (permit number AVD115002015221), and was performed in compliance with the Guide for the Care and Use of Laboratory Animals.[19]

The study was performed in 12 healthy female pigs (Van Beek SPF, Lelystad, the Netherlands), with an average weight of 60–75 kilograms. All animals were housed under standard laboratory conditions and acclimatized for 7 days upon arrival. The animals were fasted the night before the surgery. The abdominal surgery was done under deep anesthesia. Midazolam 0.7ml/kg intravenously, ketamine 13 mg/kg intravenously and atropine 0.05 mg/kg intramuscularly were applied as premedication for anesthesia and thiopental 4 mg/kg intravenously was administered for the induction of anesthesia. After premedication, midazolam 1ml/kg/hour, sufentanil 0.01 ml/kg/hour and cisatracurium 0.09 ml/kg/hour were administered.

### Surgical procedure and electroporation procedure

**Fig 1:**
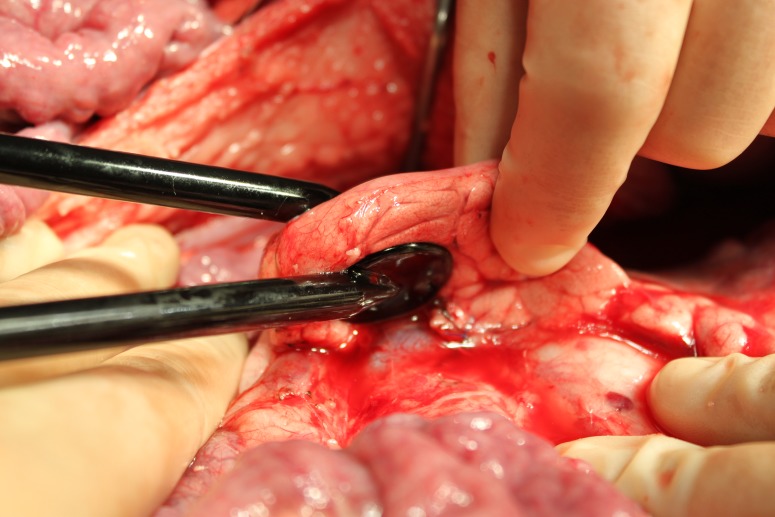
Paddles placement. Placement of paddles prior to IRE procedure.

**Fig 2:**
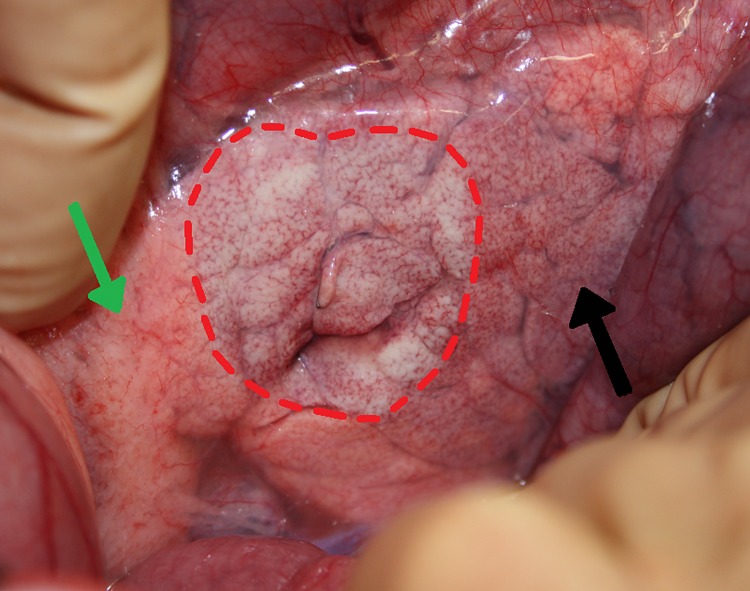
Macroscopic changes. Macroscopic image directly after IRE in situ: the red dotted line indicates the location of the paddle at the ventral side, damaged pancreatic tissue (black arrow) and undamaged pancreatic tissue (green arrow).

**Fig 3:**
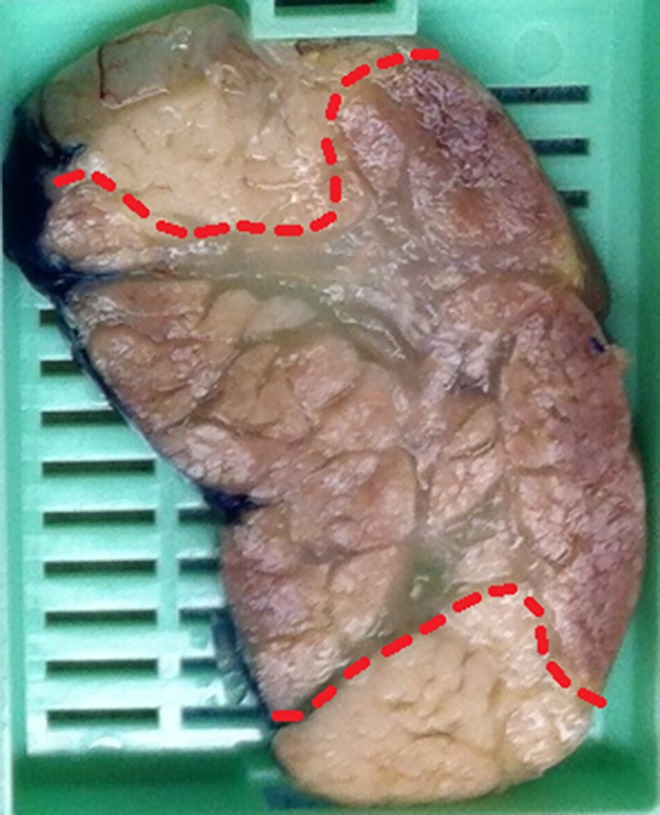
Macroscopic changes. Macroscopic image of a cross-section of the ablated area after formalin fixation: the red dotted line indicates the border of the macroscopic damaged area in the central part of this section.

A midline laparotomy was performed to expose the pancreas within the right abdomen. The pancreas was mobilized to enable positioning of the paddles. Target areas were selected which contained sufficient pancreatic tissue to completely cover the surface of both paddles. Two parallel round pediatric 25mm diameter cardiac defibrillation paddles (Inc, Redmond, WA) had been mounted on a custom fixture to ensure parallel and in-line placement around the target tissue (Fig 1). The paddles were positioned and the distance between the plate electrodes was measured with a caliper. Three different settings were investigated, based on previous performed pilot studies. To minimize the intra-individual variation, three different areas of the porcine pancreas were treated with a different method of electroporation: 1) a single ablation of 50 J, 2) a single ablation of 100 J, and 3) a series of 4 ablations of 50 J. The spacing between the ablation sites was at least 2 centimeter. Voltage and current waveforms of all applications were recorded on a storage oscilloscope to measure totally applied current and voltage. The energy source was a monophasic external defibrillator (Lifepak 9, Physio-Control, Inc, Redmond, WA). A large skin patch (7506, Valleylab Inc, Boulder, CO) on the lower back served as an indifferent electrode. The ablative area was visually inspected directly after each application to detect tissue discoloration and/or charring (Figs 2 and 3). Small stitches (Ethilon 4.0) were placed at the center, on both sides of each target area to mark the position of each paddle for histological analysis.

Literature showed necrosis of the acinar cells and sharp demarcation between viable and non-viable tissue, 2 hours after pancreatic tissue ablation, which further progressed over time.[20,21] Given the maximum duration to keep animals under anesthesia at the Laboratory of Animals was around 6 hours, this duration was maintained (Table 1). Subsequently, a total pancreatectomy was performed and the pigs were terminated by an overdose of Euthasol.

**Table 1:** Ablative characteristics.

Energy(J)	Peak power(V); Mean, SD	Peak current (A); Mean, SD	Peak resistance (Ω); Mean, SD	Tissue thickness	Electric field (V/cm); Mean, SD	Time to pancreatectomy
50	755 (134)	25.3 (3.1)	25.6 (7.5)	7.2 (4–10)	1107 (195)	6:14 (5:45–6:24)
100	1054 (190)	32.4 (5.4)	34.2 (11.0)	7.3 (4–10)	1512 (407)	6:12 (5:48–6:26)
4x 50	749 (106)	22.4 (2.9)	34.7 (9.6)	7.7 (5–12)	1016 (223)	6:15 (5:48–6.25)

V, Power reported in Volts. A, current reported in ampere. Ω, resistance in ohm. Tissue thickness in millimeters and time in hours are reported in mean and ranges.

### Histological evaluation and measurement of ablated area

The ventral part of the lesion was colored with blue ink. After formalin fixation, multiple 2–3 mm thick sagittal segments (range 2–4) of each lesion were taken at the level of the sutures. Paraffin-embedded segments were sectioned and stained with hematoxylin and eosin (H&E).

**Fig 4:**
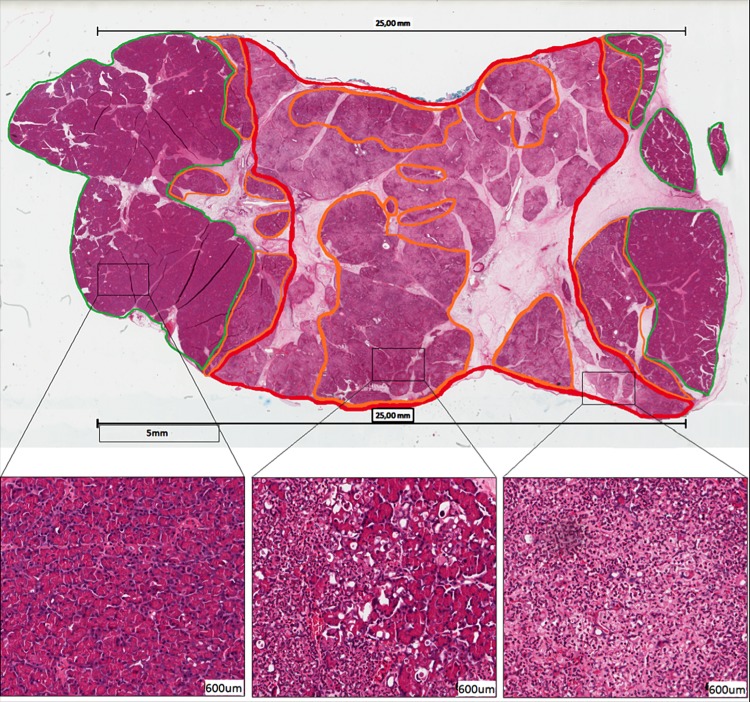
Microscopic image. Microscopic histology of porcine pancreatic tissue treated with a single application of 50 J; The location of both paddles is shown as two black bars with a length of 25 mm on both sides of the tissue. The parenchyma is marked as necrotic (red), transition area (orange); vital (green). Magnifications (H&E stain; scale bar 600 mircon.): vital pancreatic cells (left), vital cells in the upper right amid a completely necrotic area (middle) and a completely necrotic area characterized by nuclear dust.

**Fig 5:**
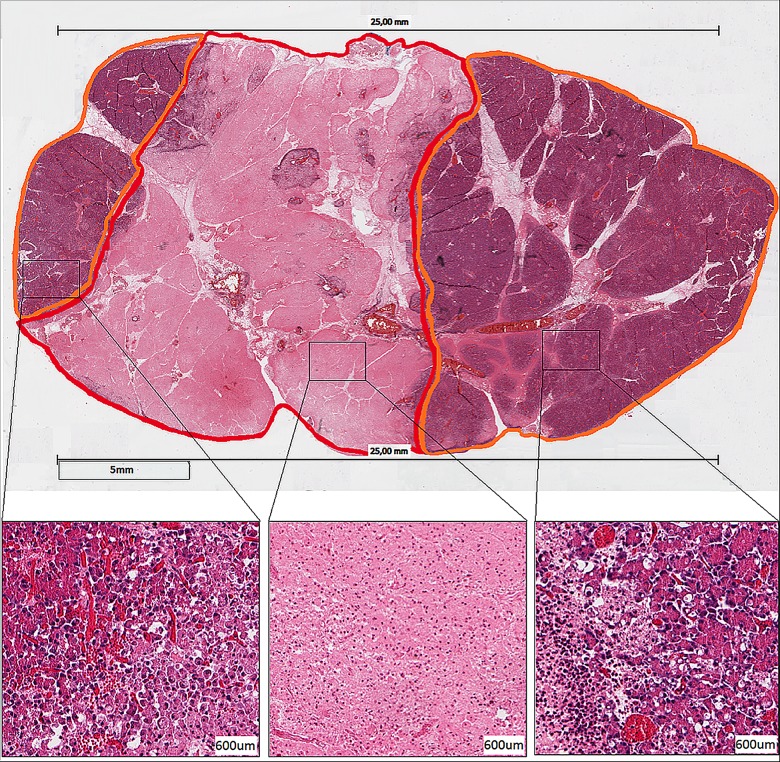
Microscopic histology of porcine pancreatic tissue treated with a series of 4 applications of 50 J; The location of both paddles is shown as two black bars with a length of 25 mm on both sides of the tissue. A homogeneous necrotic area marked red with a border zone (orange) on both sides is shown. Magnifications (H&E stain; scale bar 600 mircon.): border zone defined as a transition area consisting of vital and necrotic cells alternating (left and right) and a completely necrotic area which shows only nuclear dust.

Digitalized H&E slides from all sections were analyzed using ImageScope (Aperio Technologies). On microscopy, the tissue was scored as complete necrosis, transition area or vital (Figs 4 and 5). An area was defined as complete necrosis when no vital cells were seen; both the cytoplasm and the cell nuclei were destroyed with only nuclear dust left. These areas were demarcated with red. The transition area, demarcated with orange, was defined as an area in which vital and necrotic cells alternate or an isolated group of vital cells amid a complete necrotic area, both with many vacuoles and the influx of neutrophils. A vital area, demarcated with green, solely consisted of vital cells. A successful ablation was defined as an area of non-viable pancreatic parenchyma between the paddles. All analyses were done by two independent investigators, blinded for electroporation method (SR, WD).

### Threshold of the electric current density

**Fig 6:**
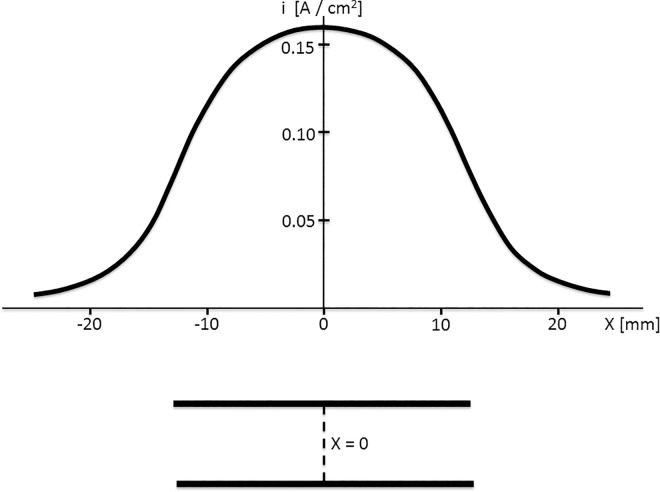
Density curve. Lower panel: A cross-section of the 2 circular paddles with a diameter of 25 mm and the plane in the middle between them (dashed line).Upper section: Current density curve in the middle plane between the 2 paddles for a tissue thickness of 7 millimeters and a nominal total ablation current of 1 Ampere.

In order to create tissue necrosis, the current density within the target tissue has to exceed a certain threshold value. This threshold value depends on the type of tissue that is ablated (i.e. liver, pancreas, prostate etc). The threshold value for the porcine pancreas, was measured at the center of the lesion between the two paddles (Fig 6 X = 0). Depending on tissue thickness (in steps of 1 mm), the nominal current density in the central plane between both paddles at a nominal delivered current of 1 ampere (A) was calculated using a semi-analytical method, in which the metal of the paddles was considered to be an ideal conductor in comparison to the surrounding tissues. Integration of the Green function of the governing differential equation over the surface of the paddles yielded an elliptic integral, which was evaluated numerically using Mathematica 7.0 software (Wolfram Research Inc., Champaign, IL, USA) (Fig 6). For each application site, this nominal current density curve was multiplied by the magnitude of the peak current as recorded during the application(s).

### Statistical analysis

Continuous variables were reported as mean and standard deviation (SD). An analysis of variance with repeated measures (RMANOVA) was applied to compare the threshold current densities between the three applications. Statistical significance was defined as p < 0.05 (2-sided). McNemar’s test was used to compare the rates of successful ablations between 50 and 4x50 or 100 J.

## Results

A total of 12 pigs with an median weight of 72 kilograms were treated. At the time of the procedure and during the 6 hours thereafter, no complications were encountered. The ablated areas were directly visible after the IRE-paddle procedures (Fig 2). The delivery of 4x50 J took approximately 20 seconds due to recording of the impulse waveforms and recharging of the impulse generator. The total IRE procedure with 3 application sites took less than 10 minutes. A mean of 3 histological sections (range 2–4) was obtained from each application site.

### Ablation

The ablative characteristics during and after the IRE procedure are displayed in Table 1. The delivered voltages and current waveforms were normal and suggested the absence of arcing, except for 2 of the total 36 ablations. During these 2 procedures, small sparks caused irregularities in the impulse waveforms and were also visible at the edges of the paddles. Both occurred during 100 J applications (pig 4 and 12) and were included in the statistical analysis. Directly after ablation, neither blood clots nor charring were seen at the application sites (Fig 2).

### Histology

Histological examination showed a successful ablation in 5/12 (50%), 11/12 (92%) and 12/12 (100%) for single application of 50 J and 100 J, and a series of 4 times 50 J, respectively, with a significantly different between 50 J and 4x 50 J (p = 0.016) as well as between 50 J and 100 J (0.031).

In addition, a difference in the content of tissue necrosis was histologically seen for the various types of applications. Each of the sections treated with 50 J contained several, large islands of transition areas amid necrotic tissue (Fig 4). After a single application of 100 J, these islands were smaller and seen only in 6/12 cases, whereas after 4 times 50 J, all sections showed a histologically homogeneous necrotic area (Fig 5). For all three applications, none of the sections showed the presence of red blood cells, suggesting the absence of vascular disruption as a consequence of the IRE procedure. In the homogenous necrotic areas, the smaller, intercalated and intralobular ducts were no longer visible, whereas the larger, interlobular ducts were still present. In the latter, ductal cell debris was seen in some cases.

### Current density threshold for lesion formation

The average current density thresholds for 50 J, 100 J and 4x 50 J were 4.3±0.8 A/cm^2^, 5.1±0.9 A/cm^2^ and 3.4±0.5 A/cm^2^ respectively. RMANOVA showed an overall significant difference between means (p = 0.027). As expected, no significant difference was found between the mean current density threshold of a single 50 J and 100 J application (p = 0.641). Therefore the mean threshold value of these two single applications together was compared to the mean threshold calculated for 4x 50 J applications, which showed a significant difference of 1.3 A/cm^2^ with 95%-CI 0.71–1.83 (p = 0.003). The current density threshold for single applications (of 50 or 100 J) was a factor 1.4 higher than the threshold for 4 consecutive applications.

## Discussion

The present study has demonstrated that IRE using paddles is feasible in a porcine model and leads to a homogeneous necrotic lesion in healthy pancreas porcine. A series of 4 applications of 50 J is found to be most effective in creating a successful ablation at an electric current density of 3.4 A/cm^2^.

The success rate in creating a necrotic lesion due to IRE-paddles was 11/12 (92%) for a single ablation of 100 J. In half of the sections at 100 J, histology showed some islands of transition areas surrounded by necrosis. It can be argued that those transition islands were in fact not vital anymore and would have gone into apoptosis eventually. Given, the effect of IRE on the ablated area progresses over time.[22] The same applies for the transition border, alongside the necrotic area. A marked deterioration in viability of the transition border over time was demonstrated by the study of Wiersinga et al, in which porcine liver was treated with RFA. [23] To address this issue, long-term survival studies are needed to demonstrate lesion maturation, determine the actual extent, homogeneity and size of long term IRE lesions.

The majority of pancreatic tumors are ductal adenocarcinomas.[24,25] Therefore, it is of great importance that both ductal cells as well as acinar cells are destroyed by IRE. At histological examination, complete necrosis of the acinar cells and disappearances of the smaller ducts was seen. However larger ducts only showed ductal cell debris in some cases. This indicates that acinar cells and the smaller pancreatic ducts can be successfully ablated by IRE, whereas the larger ducts might be resistant to the ablation. Again, long-term survival studies should determine the real, long time effects of IRE in pancreatic tumor tissue.

Comparing the use of paddles to conventional IRE, there are some advantages. The procedure time including paddle placement, was 10 minutes from end of mobilization of the pancreas until finishing the IRE procedure. This compares favourably with the procedure time of conventional IRE, which may take 1–2 hours on average.[18,26] Paddles can easily be placed around a tumor, in case the tumor can be mobilized in the retroperitoneal cavity. Presumably, in case the tumor has grown into surrounding tissue, making mobilization difficult, than placement of needles would be preferred. However, creating a sufficient electric field between the needles can be challenging with limited access to the tumor retroperitoneal. Moreover, multiple applications or the potential use of larger paddles might enable the treatment of larger tumors. Most important, we think that the use of paddles may overcome the occurrence of pancreatic fistula often observed following the conventional IRE.[17] Once more, long-term survival studies are needed in order to demonstrate this improvement.

Electric field values in Table 1 are calculated by dividing applied voltage by the distance between both paddles. These data should be interpreted with care because the electrode-tissue interface polarizes during the application. This polarization counteracts applied voltage and consequently, the voltage gradient within tissue will be smaller than applied voltage. Current density inside the tissue however, is directly proportional to applied current and therefore has more scientific relevance.

The most important limitation of this study is that the feasibility of IRE-paddles was tested in normal porcine pancreatic tissue. It is known that pancreatic cancer tissue has a significantly different structure yielding a strong desmoplastic reaction. Therefore, IRE in pancreatic carcinoma may need other settings when compared with normal pancreatic tissue.[27] Moreover, in pigs, the pancreas is located intra-peritoneal, whereas the pancreas in humans lies retroperitoneal. This retroperitoneal location entails some difficulties. The access to the tumor is limited, which might be worsened in case of tumor ingrowth in (deep) adjacent structures, making mobilization even more difficult. Furthermore, since this is not a survival study, data on outcomes such as complication and the extent of the lesion later than 6 hours are lacking. Final, small sparks between the edges of the paddles were observed during 2 of 12 applications at 100 J. This may have affected the calculated current density threshold values for these 2 corresponding lesions However, at histology examination, both sections showed complete necrosis of the ablated area.

## Conclusion

IRE performed with two parallel paddles is feasible in creating a homogeneous necrotic lesion in the porcine pancreas. Our results favor administration of a series of 4 applications of 50 J. Long-term studies are needed to investigate the safety of this technique, the homogeneity, content and size of chronic lesions with special emphasis on pancreatic fistula formation. Furthermore, IRE-paddles should be tested on pancreatic cancer tissue to ensure these settings are sufficient for the ablation of cancer tissue as well.

## Supporting Information

S1 File The ARRIVE Guidelines Checklist. (PDF)
